# Effect of an educational intervention based on the theory of planned behavior on improving medication adherence in patients with multiple sclerosis treated with injectable disease-modifying drugs: randomized controlled trial

**DOI:** 10.1186/s12889-023-15910-6

**Published:** 2023-05-30

**Authors:** Sara Hamtaeigashti, Mohsen Shamsi, Mohammad Ali Sahraian, Raheleh Soltani, Amir Almasi-Hashiani

**Affiliations:** 1 Department of Health Education, Faculty of Health, Research Committee, Arak University of Medical Sciences, StudentArak, Iran; 2 Department of Health Education and Health Promotion, Faculty of Health, Arak University of Medical Sciences, Arak, Iran; 3 Multiple Sclerosis Research Center, Neuroscience Institute, Tehran University Of Medical Sciences, Tehran, Iran; 4 Department of Epidemiology, Faculty of Health, Arak University of Medical Sciences, Arak, Iran

**Keywords:** Multiple sclerosis, Treatment adherence drugs, Educational intervention, Theory of planned behavior, Randomized controlled trial

## Abstract

**Background:**

Adherence to prescribed treatment in chronic diseases, as occurs in multiple sclerosis (MS), is a critical factor for a successful therapeutic response. This study aimed to investigate the effect of educational program based on Theory of Planned Behavior (TPB) on treatment adherence in patients with multiple sclerosis (MS) receiving injectable immunomodulatory drugs.

**Methods:**

The present study is an educational randomized controlled trial research that was conducted on 100 patients with MS who had gone to MS clinic in Tehran city (Iran). The samples were randomly assigned to the intervention (*N* = 50) and control groups (*N* = 50). Data collection instrument was a researcher-made questionnaire based on TPB. Then, educational program was performed for the intervention group through four educational sessions. After three months, data collection was repeated for the two groups and data were analyzed.

**Results:**

The knowledge and performance of the intervention group on treatment adherence drugs increased from 56.25 ± 20.3 to 78.31 ± 15.57 and 56.22 ± 5.76 to 71.62 ± 12.01 after the education respectively (*p* < 0.001). The mean of construct of TPB in the intervention group also increased after the intervention (*p* < 0.05).

**Conclusion:**

Applying the TPB model proved is very effective in developing an educational program for patients with MS, to enhance treatment adherence drugs. Besides such programs, follow-up education for controlling and monitoring are highly recommended.

**Trial registration:**

This trial has been registered at Iranian Registry of Clinical Trials, IRCT20210808052109N1. Prospectively registered at 12-Aug-2021, (12/8/2021) available at: URL: https://en.irct.ir/trial/57994

## Introduction

Multiple sclerosis (MS) is the most common progressive neurological disease in young adults and the most common non traumatic cause of disability worldwide. This disease is more common in women between the ages of 20 and 40 and is the third leading cause of disability in adults [1]. The prevalence of MS is more than 2.5 million people worldwide [2]. In Iran, the number of patients with MS has increased in recent years, and the prevalence of this disease has increased from 5 to 51 per hundred thousand people [3].

The complications of MS disease can have a devastating impact on patients' role-playing, occupational status, and daily activities, and ultimately on their quality of life. One study found that the average cost for MS patients suffering from this disease for a long time is $35,000 per year, so any program that slows the progression of this disease and helps people be independent can reduce costs [4].

Although there is no definitive cure for MS disease, but effective medications have been made available in the last decade to control the course of the disease, and these medications are known as disease-modifying drugs (DMDs) [5]. Common side effects of these medications include hypersensitivity reactions with throat tightness, swelling, rash, hives, itching, and lipoatrophy at the injection site [6]. The most common reason for discontinuation of treatment with these drugs is their side effects and treatment failure [7].

In a study meta-analysis by Giovannoni and colleagues that analysed the data of 50 randomized studies and 19 observational studies in MS, mean discontinuation rates of 17–36% for such therapies were noted [8]. In a systematic review of 24 studies published between 2001 and 2011, Menzin and colleagues reported adherence rates in patients with MS ranging from 41 to 88% [9]. Erbay et al. reported an adherence rate of 59.6% in MS patients in Turkey [10].

The definition of adherence based on WHO definition is the extent to which a person’s behavior–taking medication, following a diet, and/or executing lifestyle changes – corresponds with agreed recommendations from a health care provider [11]. Factors leading to non adherence to medication include the persistence of disease symptoms, occurrence of disease flare-ups, and side effects of medication injections. Patient education by nurses and medical staff is one of the most important aspects of increasing medication adherence [12].

For more effective training, it is better to rely on behavioral theories, which include the theory of planned behavior (TPB). According to this theory, patients perform appropriate behavior when they have a positive attitude toward the results of performing their behavior and, along with this interest, are encouraged by others as subjective norms to perform behavior for which they acquire the necessary skills and have perceived behavioral control (PBC). In this case, the behavioral intention is formed and leads to the patient's behavior [13, 14]. According to the systematic review study [15] and meta-analytic review [16] study and literature TPB is capable of explaining 20% of the variance in prospective measures of actual behavior (and 27 to 39% of intention).

Research has shown the effectiveness of educational interventions in reducing the complications of MS [17–20], but an extensive search of databases did not find information on the effectiveness of TPB-based educational interventions in relation to medication adherence in MS patients. Therefore, the aim of this study was to investigate the effect of TPB-based education on adherence of MS patients treated with first-line injectable drugs, specifically interferon beta-1a (Rebif).

## Methods

### Study design (Participants, Sample size and Sampling)

This study was conducted as an educational randomized controlled trial (single blind) on patients with MS receiving injectable immunomodulatory drugs referring to the County MS Clinic in the city of Tehran, Iran from 2021 to 2022.

Prospectively registered at 12-Aug-2021, https://en.irct.ir/trial/57994. This study adheres to CONSORT guidelines.

The sample size was calculated based on previous study Dashti et al. [17] with considering α equal to 5% and β to 0.1 using the following formula, the sample size was calculated as 40 participants in each group of control and intervention, which this number increased to 50 considering sample loss. So, the total sample size was 100.

$$n=\frac{{\left({Z}_{1-\frac{\alpha }{2}}+{Z}_{1-\beta }\right)}^{2}({\delta }_{1}^{2}+{\delta }_{2}^{2})}{{({\mu }_{1}-{\mu }_{2})}^{2}}$$

In this study having means of 35.42 and 59.51 and standard deviations of 11.36 and 9.88 for the intervention group before and after the intervention for self- care related complication in women with MS respectively in Dashti et al. [17] an effect rate of 0.6 was obtained indicating a large effect size and the same effect rate was considered for this study.

For sampling, a list of all patients was obtained from MS Clinic of hospital (3000 patients). Out of the patients having medical records in the clinic, 100 patients were selected through systematic sampling and were randomly (every other person) assigned to the intervention (*n* = 50) and control (*n* = 50) groups through random allocation rule method.

For systematic sampling after determine target sample size (100 patients), we calculated interval, k, by dividing total estimated population size (patients MS) by sample size. This can be a rough estimate rather than an exact calculation. In this way 3000 MS present in clinic and based on the sample size 100therefore our sampling interval k thus equals 3000/100 = 30.

Then selected the sample and collect data based on the list of population MS, randomly select a starting point on our list, (based on the table of random numbers, a starting point was selected for sampling) and from there, selected every kth member of the population based on inclusion criteria to include sample.

### Inclusion and exclusion criteria

Inclusion criteria were having medical records in the MS Clinic, being at the age range of 20–50 years, being literate, receiving injectable immunodulatory drugs at least one years ago and to having smart phone ownership and ability to communicate on social media (WhatsApp) (to enable data collection during the COVID-19 pandemic) also considered inclusion criteria of the participant in this study.

Exclusion criteria were change in treatment plan, unwillingness to continue cooperation, not being continuously present in training sessions and lack of availability of patients when completing the post-test questionnaire (those lost to follow up).

### Study setting (Conceptual framework)

In this study the primary outcomes was knowledge, construct of TPB (Attitude, Subjective norm, Perceived behavior control, Intention) and secondary outcome was treatment adherence behavior's.

The pre-test was administered to both groups based on the questionnaire. The intervention group received trainings based on TPB and the control group received routine cares. Then the patient were followed up for three months. After that the post-test was administrated and finally the effect of education on treatment adherence in patients with MS receiving injectable immunomodulatory drugs was re-evaluated.

According to the similar study and panel of experts, three months of follow-up was considered sufficient time to establish consistency, stability, and sustainability in treatment adherence in patient with MS receiving injectable immunomodulatory drugs [17, 21].

The conceptual framework of conducting this study was that according to the study criteria, the samples were selected by referring to the MS clinic and divided into control and intervention groups. Then pretest was administered to both groups based on the questionnaire and the intervention group received training based on the TPB.

By utilizing the attitude construct, patients are firstly exposed to non adherence drugs problem and understand the complications and at the same time they are taught the consequence for advantage and disadvantage of treatment adherence drugs. Then by utilizing the subjective norm and increasing patients' perceived behavior control, they were taught behaviors related to treatment adherence drugs. The control group also received routine MS clinic training. Then the patients were followed for three months and then posttest was administrated and the effect of education on their treatment adherence drugs was re-evaluated.

**Fig. 1:**
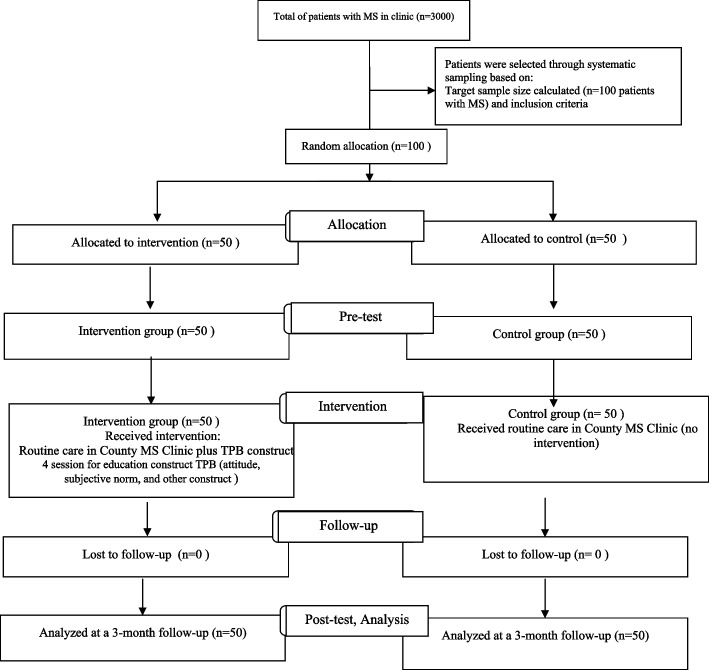
Flow diagram of the participants. From a total of 3000 MS patients referring to MS clinic, 100 were selected with systematic sampling based on the inclusion criteria and then randomly divided into intervention and control groups (50 each). In both groups, pre-test was administrated and then the intervention group received TPB based education and the control group received routine training. Three months later, post-test was administrated in both groups and treatment adherences drugs were compared in both groups

Figure 1 shows the framework and flow diagram of the participants during the study period.

### Data collection and scoring

The data collection tool in this study was a valid and reliable researcher-made questionnaire consisting of questions on demographic information, knowledge, constructs of the Theory of Planned Behavior and performance in treatment adherence drugs in patients with MS. In this study higher score indicate higher level of knowledge, attitude, subjective norms, perceived behavior control and performance of treatment adherence drugs in patient with MS.

Data were collected in the following sections:

1. **Demographic variables and clinical characteristics:** Patient-related factors were age, sex, marital status, family environment (alone or with others living), educational level, monthly income level based on the USD $), movement restriction, Duration use an injectable immunodulatory drugs and memory disorders was also included.

2. **Knowledge questionnaire:**

The MS patients’ knowledge of treatment adherence drugs questionnaire consisted of 8 multiple-choice questions.

For the knowledge the correct answer scored 1 and the wrong one scored 0 and the total score of awareness was calculated and reported between zero and 100.

In this study, based on the opinion of the expert panel (include multiple sclerosis and related disorders specialist, neurology specialist, general physician, health education and health promotion specialist, nurse expert in MS and one specialist in epidemiology) with considering to the cut-off point, the level of knowledge was divided and reported into three categories: poor knowledge (0–30 scores), moderate (31–60 scores) and good knowledge (over 60 scores).

3. **Theory planned behavior construct questionnaire:**

The TPB constructs questionnaire on treatment adherence drugs behaviors consisted of attitude (8 questions), subjective norms (7 questions), perceived behavior control (7 questions), intention (4 questions).

The questions of the TPB constructs were scored on a 5-point scale from strongly agree, agree, no idea, disagree and strongly disagree scoring 1 to 5.

Therefore the scores range of each theory construct for understandable was finally calculated and reported between one and 100.

4. **Performance questionnaire:**

The performance questionnaire on treatment adherence drugs also consisted of 13 questions that assessed over the recent 2 week.

The questions of performance treatment adherence drugs questionnaire were scored on a 5-point scale of behavior evaluation from never, rarely, sometimes, often and always scoring from 0 to 4 and the scores in this part were reported between 0 and 100.

According to Ajzen (who is one of the founders of the theory of planned behavior), using the self-report method and relying on individual’s reports rather than direct observation and immediate measurement of the target behavior, due to some problems in obtaining data in the time limits, is an accepted method in TPB studies [22, 23].

In this study, based on the opinion of the expert panel with considering to the cut-off point, the treatment adherence drugs of MS patients was divided and reported into three categories: poor adherence (0–30 scores), moderate adherence (31–60 scores) and good adherence (over 60 scores).

### Validity and reliability of questionnaire

The development of the TPB in patients with MS a questionnaire intended to an explain patient choices to use or not to use treatment adherence drugs was based on the instructions given by Ajzen [24]. We focused on assessing all relevant awareness, cognitive and emotional issues (belief composites) presumably processed by MS-patients considering a performance about TAD. The original pool of about 85 awareness and belief composites was generated by extracting salient statements from interview with 30 physician patient consultations on TAD performance. Statements identified as relevant were classified according to their underlying constructs in terms of the TPB framework. They were allocated to knowledge and one of the three main domains TPB (attitude, subjective norm, and perceived behaviour control).This pool of knowledge and belief composites and their allocation were discussed and supplemented by an expert panel of three multiple sclerosis and related disorders specialist, two neurology specialist, one general physician, two health education and health promotion specialist, one nurse expert in MS and one specialist in epidemiology.

The conceptual framework of the validity and reliability of this study was that according to the similar study Kasper et al. [25] about developing, validity and reliability questionnaire based on TPB. During this process, based on a study Kasper et al. [25] classification of the statements was checked and about 8 new statements were added from nurse and physicians’ clinical practice. In the next step, based on the underlying concept items were created to exhaustively cover the TPB framework for the scenario of performance TAD. With regard to uniqueness, similarity and disjunctiveness, primary instrument were piloted with 15 MS patients in the MS outpatient clinic to check comprehensibility and relevance. The final questionnaire consisted of 47 items including Knowledge, construct of the TPB domains and the performance questionnaire on treatment adherence drugs.

The validity and reliability of this questionnaire was approved and it was completed before the training intervention and three month after the intervention by both control and intervention groups. In this tool, those questions with Content Validity Ratio (CVR) score higher than 0.62 and Content Validity Index (CVI) score higher than 0.79 were considered as appropriate and included in the study [26].

To verify its reliability, the questionnaire was given to 30 MS patients and its reliability was calculated as upper 0.8 using Cronbach’s alpha.

### Educational intervention

Before performing the training intervention and in pre-test stage, the questionnaires were completed by both groups and entered the computer to be used for determining the training needs and the constructs to be presented in training sessions. Then, according to the TPB and based on the results of the needs analysis, the training program was prepared for four 60 to 120-min sessions at an interval of one week for 1 month (one session per week) for targeted at the intervention group. With considering outbreak Covid-19 and vulnerable MS patients for morbidity to Covid-19 disease therefore three of session training implementation through social network. The materials were presented in the sessions through lectures, question and answer, slide and film presentation, to benefit all the time in class and make the training available for further study by the patients.

In the first training intervention sessions, knowledge of MS patients was emphasized aiming at gaining an appropriate knowledge of MS and factors affecting with considering treatment adherences drugs deterioration and acceleration of MS complications, the second session's focus on; attitude and subjective norm was touched by presenting the statistics on prevalence of problems resulted from MS and vulnerability of patients to disease complications resulted from inappropriate treatment adherence drugs. Moreover the materials of these sessions emphasized on the benefits resulted from performing treatment adherences drugs for ex. reduced MS complications, reduced need to clinic services and lower medical expenses, feeling of calmness and internal joy. Subjective norm affecting performing treatment adherences drugs including neurologist, physicians, MS clinic nurses, family members, television and other MS patients were emphasized.

The third session's focus on perceived behavior control and intention; perceived behavior control construct was emphasized by empowering the patients by the aim of facilitating performance of treatment adherences drugs through presenting educational film and images on slides, use of reminder for adherences drugs (dosage, time and kind of drugs based on treatment of plan), adherences to protocol treatment recommended physician and providing them with booklets and pamphlets.

The fourth session's focus on performance of treatment adherences drugs; behavior construct was emphasized to facilitating performance of treatment adherences drugs through presenting educational film for technique injection, images for titer drugs, practical training for injection correct.

In this study control group received only routine care includes visits at a clinic from a doctor or nurse, an educational for only injection drug in appropriate tissue.

During the follow up for reminder, the educational content included voiced PowerPoint, short educational messages, educational photos with short explanations, and short educational videos that were sent to the MS patients of the intervention group. Moreover in this time group discussion and question and answer were conducted to better understanding of the training contents and feedbacks were provided; each patients was encouraged to ask questions.

Three months after the training intervention, the questionnaire was given again to the both groups and the all 100 patients completed them.

### Statistical analysis

The data were analyzed using SPSS version 20.0 (IBM Corporation, Armonk, NY, USA) through descriptive statistics (Mean, SD, frequency and percent) and inferential statistics (including independent t-test, paired t-test, Chi-square, Fisher exact test). To investigate the normality of the data, Kolmogorov–Smirnov test was used and normal distribution of the data was obtained. However concerning the gender, marital status, level of education and memory disorders difference between MS with regard to treatment adherence drugs and also due to the small size of the groups, the distribution of data was non normal and therefore nonparametric tests (Wilcoxon, Mann–Whitney and Kruskal–Wallis terst) were used.

### Ethical considerations

Written informed consent was obtained from all the participants. Moreover, after the study, the training materials such as the booklets were given to the control group.

The present study was approved by ethics committee of Arak University of Medical Sciences (code: IR.ARAKMU.REC. 1400.082) and registered in Iran Registry Clinical Trials (code: IRCT20210808052109N1).

## Results

### Sample characteristics

Results of the study showed that the mean and standard deviation (SD) of age among multiple sclerosis patients in the intervention and control groups were 33.82 ± 10.84 and 33.24 ± 9.25 years respectively (*p* = 0.774).

The two groups were comparable with respect to demographic and disease related characteristics. In both groups, the differences in sex, educational status, marital status, health insurance, income level, movement restriction, living alone and duration of drugs use were not significant and they were nearly the same in terms of variables (Tables 1).

**Table 1:** Comparison of the intervention and control groups, concerning the sociodemographic and clinical characteristics variables

Group Variable	Control		Intervention		*p*- Value
	Number (N)	Percent (%)	Number (N)	Percent (%)	
Sex^a^					
Female	41	82	35	70	0.121
Male	9	18	15	30	
Heath insurance ^b^					
Yes	44	88	46	92	0.370
No	6	12	4	8	
Marital status^b^					
Married	25	50	28	56	0.417
Single	24	48	18	36	
Divorced	1	2	3	6	
Educational qualification^a^					
Elementary	10	20	10	20	0.620
Diploma	9	18	16	32	
University	31	62	24	48	
Monthly income level ^a^					
Weak (< 250 USD $)	5	10	1	2	0.227
Medium (251–500 USD $)	20	40	20	40	
Good (> 500 USD $)	25	50	29	58	
Movement restriction ^b^					
Yes	0	0	0	0	1
No	50	100	50	100	
Living alone ^a^					
Yes	6	12	5	10	0.50
No	44	88	45	90	
Memory disorders ^b^					
Never	12	24	15	30	0.67
Occasionally	17	34	14	28	
Frequently	8	16	9	18	
Usually	10	20	7	14	
Always	3	6	5	10	
Receive previous education ^a^					
Yes	17	34	12	24	0.189
No	33	66	38	76	
Duration use an injectable immunodulatory drugs^b^					
Less than 2 month	15	30	24	48	0.220
Three month	3	6	8	16	
Fourth month	2	4	1	2	
Five month	1	2	0	0	
Six month	2	4	0	0	
Between 6 to 12 month	17	34	8	16	
Between 1 to 2 years	6	12	7	14	
Between 2 to 3 years	3	6	2	4	
Between 3 to upper 4 years	1	2	0	0	

^a^ Chi-square Test

^b^ Fisher Exact Test

### Evaluation of intervention

The difference between two groups has been shown in Table 2. Comparison of the baseline mean scores showed no significant difference in knowledge (*p* = 0.132), subjective norm (*p* = 0.516), perceived behavior control (*p* = 0.185), behavioral intention (*p* = 0.189) and treatment adherence drugs (*p* = 0.184) between the two groups (Table 2).

**Table 2:** Comparison of the intervention and control groups, concerning the TPB, before and after the intervention

Group Variable	Intervention		Control		*p*-value^a^
	**Mean**	**SD**	**Mean**	**SD**	
**Knowledge**					
**Before**	56.25	20.3	62.75	22.3	0.132
**After**	78.31	15.57	64.28	18.39	0.001
***p*-value**^**b**^	0.009		0.714		
**Attitude**					
**Before**	70.40	21.55	75.40	21.49	.0248
**After**	93	16.9	81.39	17.31	0.001
***p*-value**^**b**^	0.001		0.2		
**Subjective norm**					
**Before**	73.88	10.76	75.29	10.90	0.516
**After**	82.62	8.97	82.51	7.96	0.946
***p*-value**^**b**^	0.011		0.035		
**Perceived behavior control**					
**Before**	73.54	11.74	77.11	14.83	0.185
**After**	87.48	9.37	80.36	15.08	0.006
***p*-value**^**b**^	0.001		0.141		
**Behavioral Intention**					
**Before**	83.50	11.83	86.50	10.79	0.189
**After**	93.90	7.97	88.20	9.83	0.002
***p*-value**^**b**^	0.004		0.65		
**Treatment adherence drugs**					
**Before**	56.22	5.76	57.5	4.30	0.184
**After**	71.62	12.01	60.56	5.26	0.001
***p*-value**^**b**^	0.002		0.21		

^a^ Independent t-test

^b^ Paired t-test

The results of paired sample T-tests showed no significant difference in the pretest and posttest knowledge scores of the control group (*p* > 0.05). The pretest–posttest mean differences of the TPB constructs were not also statistically significant in the control group (*p* > 0.05), except for the mean difference of the subjective norm that showed a significant difference upon completion of the study (*p* = 0.035).

The mean knowledge score of TAD was 56.25 ± 20.3 before the education and 78.31 ± 15.57 after the implementation of intervention in the intervention group. The difference between pre intervention and post intervention mean knowledge scores was statistically significant (*p* = 0.009). The paired T-test of health belief scores showed that the scores of the health belief subscales TPB were mostly higher after the intervention when compared with before the intervention (*p* < 0.05) in the intervention group.

Independent T-test showed that the mean score of knowledge, attitude, subjective norm, behavioral control, behavioral intention and patients performance in relation with the adherence drugs in patients three months after the educational intervention, the difference was statistically significant (*p* < 0.05). The greatest increase was seen for the attitude scores; the pre-test average was 70.4 ± 21.55 and increased to 93 ± 16.9 after the intervention.

So that the performance of treatment adherence drugs in the intervention group after the education increased from 56.22 ± 5.76 to 71.62 ± 12.01 (*p* = 0.002). The difference between two groups has been shown in Table 2.

Concerning the gender difference between MS men and female with regard to treatment adherence drugs, the results showed that in the intervention group, the adherence score of the MS men and female significantly increased and this promotion in female is from 57 ± 5.3 to 70.94 ± 11.78 (*p* = 0.01) after the intervention. Comparison of the intervention and control groups, concerning the performance of treatment adherence drugs based on marital status, level of educational and memory disorders, before and after the intervention showed in Table 3.

**Table 3:** Comparison of the intervention and control groups, concerning the performance of treatment adherence drugs based on some demographic and clinical characteristics variables, before and after the intervention

**Group Variables**	**Intervention *N* = 50 (Female = 35, Male = 15)** **Mean** ± SD		***p*-value**^**a**^	**Control, *N* = 50** **(Female = 41, Male = 9)** **Mean** ± SD		***p*-value**^**a**^
	**Before intervention**	**After intervention**		**Before intervention**	**After intervention**	
**Sex**						
**Female**	57 ± 5.3	70.94 ± 11.78	0.01	57.39 ± 4.55	60.64 ± 5.63	0.145
**Male**	54.4 ± 6.3	73.2 ± 12.79	0.01	58.44 ± 2.92	60 ± 2.69	0.170
***p*-value**^**b**^	0.227	0.385		0.161	0.438	
**Marital status**						
**Married**	57.07 ± 5.23	71.71 ± 12.70	0.01	57.44 ± 4.60	59.88 ± 4.30	.243
**Single**	55.26 ± 6.82	72.10 ± 11.63	0.01	58.04 ± 3.81	61.20 ± 6.22	0.118
***p*-value**^**b**^	0.310	0.148		0.563	0.788	
**Educational qualification**						
**Elementary**	51.35 ± 5.48	66.8 ± 9.63	0.002	53.2 ± 5.1	55.2 ± 5.5	0.684
**Diploma**	57.2 ± 6.12	70.3 ± 10.2	0.001	56.9 ± 5.8	59.7 ± 4.8	0.835
**University**	61.3 ± 6.5	81.21 ± 9.14	0.001	60.32 ± 5.7	63.2 ± 5.3	0.441
***p*-value**^**c**^	0.041	0.002		0.112	0.326	
**Memory disorders**						
**Never**	59.10 ± 6.1	78.29 ± 11.41	0.001	63.30 ± 5.3	66.1 ± 5.78	0.361
**Occasionally**	57.23 ± 5.4	74.52 ± 10.48	0.001	59.83 ± 4.9	62.44 ± 6.1	0.265
**Frequently**	54.36 ± 4.8	68.67 ± 11.15	0.001	52.43 ± 5.8	56.3 ± 4.3	0.643
**Usually or Always**	53.94 ± 5.7	67.93 ± 12.70	0.001	52.1 ± 4.4	54.9 ± 5.1	0.421
***p*-value**^**c**^	0.058	0.079		0.162	0.0911	

^a^Wilcoxon test

^b^Mann-Whitney test

^c^Kruskal-wallis test

Pre-intervention evaluation of knowledge revealed that the only 34% of the MS in intervention group had good knowledge regarding TAD that an increased to 76% after intervention with significant difference (*p* = 0.01). Comparison of the intervention and control groups, about knowledge and performance of treatment adherence drugs, before and after the intervention showed in Table 4.

**Table 4:** Comparison of the intervention and control groups, concerning the knowledge and performance of treatment adherence drugs, before and after the intervention

Group Variables	Intervention *N* = 50 N(%)		Control, *N* = 50 N(%)	
	Before intervention	After intervention	Before intervention	After intervention
**Knowledge**				
**Poor**	7(14)	0	6(12)	3(6)
**Moderate**	26 (52)	9 (18)	20(40)	24 (48)
**Good**	17 (34)	41 (76)	24(48)	22 (44)
***p*-value**^**a**^	0.01		0.254	
**Treatment adherence drugs**				
**Poor**	5(10)	0	4(8)	1(2)
**Moderate**	27 (54)	11(22)	25 (50)	27 (54)
**Good**	18 (36)	39 (78)	21 (42)	22 (44)
***p*-value**^**a**^	0.01		0.141	

^a^ Fisher Exact Test

## Discussion

The results of this an educational randomized controlled trial study showed that an improvement of patients in medication adherence was due to the use of theory-based education methods combined with active follow-up.

After the educational intervention, patients' knowledge of medication adherence increased significantly. Education on a better understanding of the medication adherence program, the proper amount and timing of medication use, symptoms of underuse or overuse of medication, as well as the provision of materials in the form of brochures and health messages, could prove effective in increasing patient awareness.

The study by Zimmer et al. on the effect of the nurses’ education program for MS patients increased their awareness of fingolimod treatment [27]. Moreover, Goldoust et al. study is in agreement with the present study in terms of improvement in the information and awareness of MS patients regarding stress management [18].

The improvement in the attitude of patients in the intervention group regarding medication adherence outcomes was due to the educational intervention programs. With emphasis on factors such as the increase in treatment costs and the occurrence of disease complications, disease progression, hospitalization, and the increase in disease flare-ups. Educational videos, pictures, and presentation of patients' experiences in the form of group discussions were used to improve patients' attitudes. In a study on medication adherence and clinical outcomes from MS, it was shown that patients with complete medication adherence had a lower rate of hospitalization for MS [28].

In the current study, to improve subjective norms, the influential role of other people who influence patients' decisions, such as physicians, nurses, and family members, was used. In addition, physicians and nurses participated in the educational sessions to educate patients, and educational materials in the form of brochures, booklets, and educational videos were also provided to family members; therefore, all these factors improved the subjective norm of the patients. Moreover, Burke et al. suggested that MS nurses, as trained healthcare professionals, played an important role in educating patients about the nature of the disease and increasing medication adherence [29]. Zimmer et al. [27] and Dhib Jalbut et al. [30] indicated that the support of nurses for medication adherence was greater than that of physicians in MS patients, which was due to nurses' greater interaction with patients.

One of the most important parameters for medication adherence in MS patients is to empower them and increase their PBC. In the present study, patients' PBC was promoted by emphasizing factors such as a better understanding of the nature of the disease by the patient to ensure adherence to treatment instructions, creating internal motivation independent of external incentives, and adherence to medication under conditions of travel or preoccupation.

In the study of Mohr et al. on MS patients who had just started interferon treatment, it was revealed that the inability of patients to inject themselves with the drug was directly related to discontinuation of the drug during the first 6 months of treatment [31].

When a medication regimen requires frequent injections, empowering patients to self-inject reduces unnecessary dependence on others or the need to visit the clinic frequently to receive medications and increases medication adherence. According to the Diagnostic and Statistical Manual of Mental Disorders of the American Psychological Association, phobia (fear) of injections is one of the main problems of MS patients and causes them not to adhere to the medication [32]. In general, the prevalence of injection phobia in the general population is estimated to be between 7% and 22% [31, 33, 34].

In a review study showed that the most frequent cause of stopping treatment is perceived lack of efficacy, and that most withdrawals from treatment occurred during the first year [35]. This information illustrates the importance of patient education and the implementation of strategies to promote adherence, which include setting explaining the critical role of adherence in treatment outcomes, recognizing and addressing barriers to optimal adherence, advocating for patients by assisting with reimbursement, identifying available resources, and involving family members and loved ones [36].

By educating patients and improving awareness, attitude, and PBC, a positive intention to adherence to medication is formed in patients. When patients have the necessary injection capabilities and skills, intention becomes behavior. In a study, patients' intention was found to be important in behaviors about stress management in MS patients [18].

Costello et al. in a study showed that patients with MS require education that clearly explains the facts regarding the possible course and nature of the disease, its symptoms, and the importance of diagnostic tests [36]. The findings of our study are consistent with the results of other studies that showed implementation of educational programs leads to improvement of behaviors among patients with MS [18–20, 34]. Similarly, Golan et al. [37] reported that an electronic notebook through smartphones could improve treatment adherence amongst MS patients.

In our study adherence in baseline in intervention and control group was 36% and 42% respectively. Our results were superior to those described by Hansen et al. [38], who showed an adherence of 30–40%, although lower than those reported by other researchers, which varied between 59.6% and 76.4% in different studies [39–41].

The improvement in patient performance on medication adherence was due to the improvement in other constructs of the TPB. In this regard, the educational intervention program emphasized teaching patients about adherence to dosage, timing of injections, and taking medications with them when traveling, as well as following the recommendations of treatment staff through educational videos and hands-on demonstrations.

Regarding the need for medication adherence in MS patients, clinical trials have demonstrated that interferons with high doses and more frequent injections (e.g., Rebif with thrice-weekly injections) are more effective than interferons with less frequent injections (e.g., Avonex with once-weekly injections), and the same repeat of injection increases the importance of medication adherence in these patients [42].

In the present study, adherence drugs was reported significant difference based on the gender, marital status, level of education and memory disorders after an intervention. So it seems that beside educational program based on TPB at the same time during outbreak Covid-19 patients are sensitive to their health and the rate of their participation in health programs and their acceptance and practice of health behaviors such as treatment adherence drugs.

In addition to the role of education for behavioral change, environmental factors should be considered. For example, patients' limited mobility and living alone can not to access medicine and visits to doctors and clinics, forgetting to take medicines and not being reminded by others in patients who live alone, or lack of help in injecting medicine by others. This factors can be effective on the level low of treatment adherence drugs in MS patients. In a study Treadway and et al. showed that the most common reason MS patients for missing injection was that they simply forgot to administer the medication, hope, depression, and support were also assessed in relation to adherence [43].

In a clinical trial study, self-care training was suggested as a useful and effective intervention to improve the performance of MS patients [44]. In Dashti et al.'s study, improving self-care in female patients with MS reduced complications caused by MS such as muscle cramps, fatigue, constipation, and forgetfulness after the implementation of the educational program [17].

Furthermore, Jones et al. found that the role of support services and patient follow-up influenced medication adherence [45]. In the present study, patients' questions and doubts were answered by communicating with them, and patients were actively followed up during the intervention and follow-up periods.

### Strengths and limitations

One of the strengths of this study was the use of various teaching methods and the combination of face-to-face and virtual training, as well as the random selection of samples.

Moreover the design of the educational intervention for increase of adherence drugs in patients with MS was based on a the need assessment (pre-test) and the constructs of the TPB, as well as on following up of the adherence drugs of patients three months after the educational intervention. Another strengths of this study is showed that how theory can be used to inform the design of an effective intervention and to guide its evaluation. However, since the TPB itself is generic and its domains are quite elementary, but it has great potential for developing specific applications for other health behavior support contexts as well.

Several limitations in this study should be noted. Firstly limitations of the study were the impact of the COVID-19 outbreak and the virtual delivery of some training due to the susceptibility of MS patients to COVID -19.

Secondly, we do not have direct clinical data related to adherence provided by physicians (e.g., medical reasons, therapeutic failure, progressive forms, adverse effects). Therefore in this study does not allow generalization of the results to other groups of MS patients because others factors may not have been fully controlled.

Third, the selection of TPB out of a pool of more than 30 theories of behaviour change [46] can be challenged. As well as rational reasoning, environmental factor for example access to equipment, physician, outbreak Covid-19 during study, costs and emotional processes also affect on a specific behavior [46, 47].

Another study limitation was selecting the samples within the limit age range of 20–50 years. Finally, in this study we are using a self-reported questionnaires. This limitation was resolved by allocating sufficient time and explicit expression of the objectives of study, and gathering information along with interviewing. Also, we followed up the patients with MS for 3 months as the longer follow up may lead to more accurate outcomes. In addition based on the recommendation of Ajzen (who is one of the founders of the theory of planned behavior), using the self-report method and relying on individual’s reports rather than direct observation and immediate measurement of the target behavior, due to some problems in obtaining data in the time limits, is an accepted method in TPB studies [22, 23].

Despite these limitations, this study provides an initial approach to the potential variables (construct of TPB) that could affect adherence to treatment in patients with MS and this warrants further study.

## Conclusion

The results of the present study showed that the design and implementation of the TPB-based medication adherence training program could produce a significant difference in the level of knowledge, attitude, subjective norms, behavioral intention, PBC, and performance of the patients in the case group.

In the current study, by increasing patients' attitudes toward medication adherence outcomes, increasing their perceived ability in this regard, and simultaneously using the effect of subjective norms (physician, nurse, and family) along with patients' active follow-up, a significant increase in the average performance of adherence.

This result should be considered by the healthcare professional when training educational program and evaluating the risk of non-adherence with therapies in patients with MS. It is essential to implement efforts to improve treatment adherence, disease care, and patients’ quality of life and to reduce public health costs for MS patients.

However, whether patient’s behavioral changes (promotion TAD) directly have clinical implications for the prevention of adverse out come and complication of MS, requires further studies. In other words, more evidence is needed to show that TPB can actually be effective in decrease outcome of disease in patients with MS. It is suggested that future studies be designed to use laboratory tests in addition to questionnaires to assess the (worse or better disease) impact of health behaviors.

## Data Availability

The datasets analysed during the current study available from the corresponding author on reasonable request.

## Abbreviations

MS: Multiple Sclerosis
TPB: Theory of Planned Behavior
TAD: Treatment Adherence Drugs
PBC: Perceived Behavior Control
CVR: Content Validity Ratio
CVI: Content Validity Index
SD: Standard Deviation
